# Thiostrepton induces apoptotic cell death at the level of BCL-2/CED-9 in *C. elegans*

**DOI:** 10.1038/s41598-025-09446-5

**Published:** 2025-07-08

**Authors:** Alanoud Al-Kaabi, Tayyiba Akbar Ali, Mahmoud Izadi, Kirti S. Prabhu, Shahab Uddin, Ehsan Pourkarimi

**Affiliations:** 1 Division of Genomics and Translational Medicine, College of Health and Life Sciences, Hamad Bin Khalifa University, Qatar Foundation, Doha, 34110 Qatar; 2 Division of Biological and Biomedical Sciences, College of Health and Life Sciences, Hamad Bin Khalifa University, Qatar Foundation, Doha, 34110 Qatar; 3 Translational Research Institute, Academic Health System, Hamad Medical Corporation, Doha, 3050 Qatar

**Keywords:** Cancer, Thiostrepton, Apoptosis, BCL-2/CED-9, *C. elegans*, ROS, Apoptosis, Target validation

## Abstract

Thiostrepton, a thiopeptide antibiotic, has been attracting increasing attention for its anti-proliferative and anti-cancer properties in various tested cell culture models. Extensive *in vitro* analysis has been conducted to understand its anti-cancer effect across multiple cancer types and cell lines, with numerous reports indicating that thiostrepton can inhibit cancer cell proliferation and tumor growth and induce apoptosis *in vitro*. On the other hand, the *in vivo* anti-tumor effect of thiostrepton remains elusive. In this study, we aimed to genetically and physiologically characterize the effects of thiostrepton on apoptosis induction *in vivo* using *C. elegans*. Our data demonstrate that thiostrepton induces apoptosis in *C. elegans*, and this apoptotic induction is independent of the genomic instability and is not related to p53 activity. Rather, the apoptotic cell death mediated by thiostrepton treatment occurs at the level of the BCL-2/CED-9 protein at the core apoptotic machinery. Furthermore, we have unlinked the high ROS (reactive oxygen species) induction reported in earlier *in vitro* studies from apoptosis induction upon thiostrepton treatment in *C. elegans*. Overall, our genetic data indicate that apoptosis induction mediated by thiostrepton occurs at the level of the core apoptotic machinery.

**Supplementary Information:**

The online version contains supplementary material available at 10.1038/s41598-025-09446-5.

## Introduction

In the last decade, a novel class of naturally occurring thiopeptide antibiotics characterized by their sulfur-containing heterocyclic rings has emerged. Thiopeptides are distinct from other natural products derived from peptides containing azoles due to a set of shared properties. Their defining property is a central six-membered ring containing nitrogen, which can exist in various oxidation states^1,2^. Among these thiopeptide antibiotics is Thiostrepton (Thio), which was first derived from *Streptomyces azureus* in 1954^3^. Historically, research on Thio focused on its antibacterial properties^4^, highlighting its role as a potent blocker of prokaryotic translation by binding to 23S rRNA and the N-terminal domain of the ribosomal uL11 protein^5,6^. The anti-cancer effect of Thio was initially detected in a small molecule screening to hunt for forkhead box M1 (FoxM1) inhibitors^7^. It is well established that the oncogenic transcription factor FoxM1 is among the most upregulated genes in various carcinomas^8-10^. FoxM1 is a member of the forkhead/winged helix protein family that acts as a transcription factor in various physiological activities, including cell cycle progression, angiogenesis, and apoptosis^11^. Importantly, treating cancer cell lines with various thiazole antibiotics, such as Thio, specifically blocks FoxM1 transcriptional activity and expression without affecting the general transcriptional profile of the cancer cells^12^. Recent emerging data strongly suggest that the various thiazole antibiotics, such as siomycin A and Thio, stabilize proteins like p53, Mcl-1, and p21^12^. More importantly, it is now evident that the downregulation of FoxM1 mediated by Thio induces apoptotic cell death in various cancer cell lines, such as MCF7 breast carcinoma cells, lung cancer, melanoma, and colorectal cancer^13-16^. A more recent study has clearly linked FoxM1 downregulation by gene silencing or its inactivation using Thio to cell cycle arrest and, subsequently, caspase-dependent apoptosis induction in the B-precursor acute lymphoblastic leukemia cells^11,16^. Some studies have reported that Thio induces apoptosis through both p53-dependent and -independent mechanisms. Thio significantly reduces cancer cell migration, invasion, and angiogenesis, and hinders tumor growth and metastasis^15,17^. While there have been extensive *in vitro* studies on the anti-cancer effect of Thio, the effect of Thio in an *in vivo* setting is yet to be characterized. In this study, we have used *Caenorhabditis elegans (C. elegans)* as an *in vivo* model system to investigate the effect of Thio on organismal development, its role in apoptosis induction, and its potential in activating DNA damage response at an organismal level.

## Results

### Thiostrepton treatment induces apoptosis in *C. elegans* germline

**Fig. 1:**
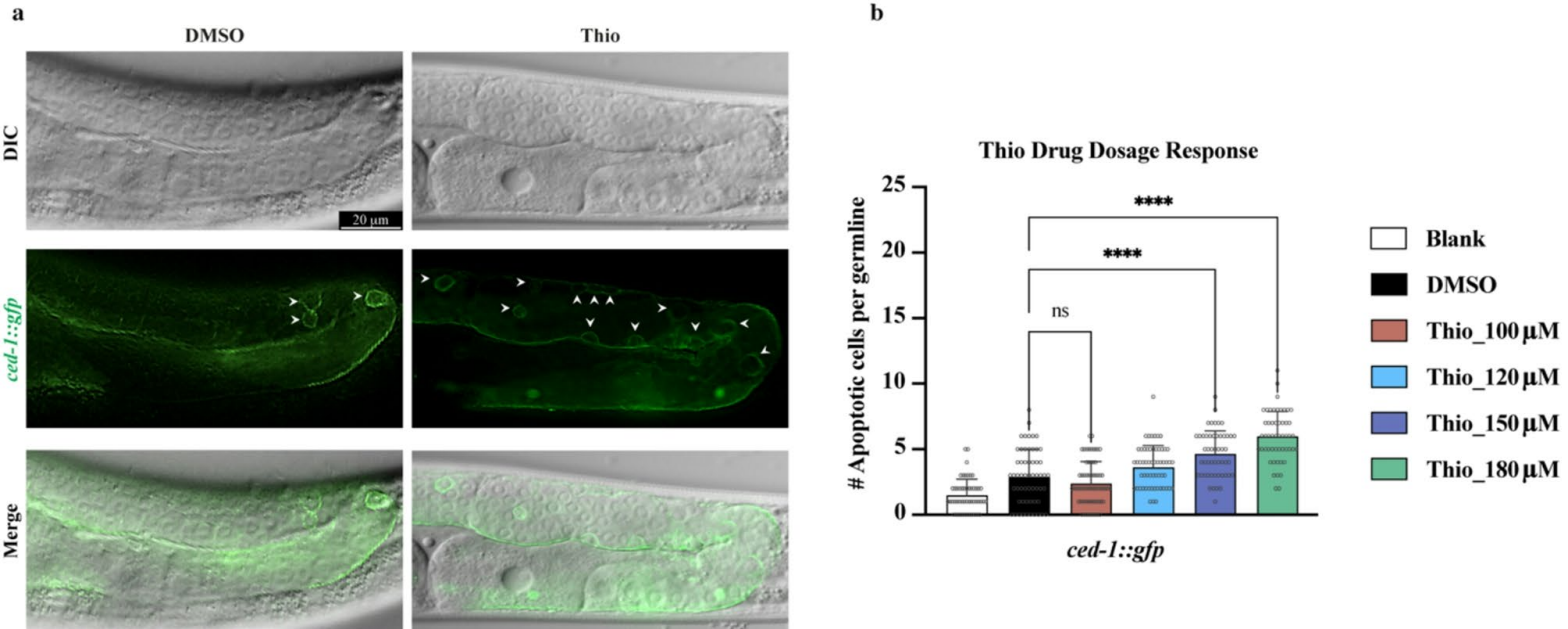
Thio Dosage Response in *ced-1::gfp* Strain. **a** Representative DIC, GFP, and merged fluorescence images of *ced-1::gfp* germlines treated with (DMSO) or 180 µM Thio for 24 h. Arrowheads indicate apoptotic cell corpses labeled by CED-1::GFP accumulation in the pachytene region of the gonad. **b** Quantification of apoptotic germ cell corpses per gonad across increasing Thio concentrations. Bars represent mean ± SEM. Statistical analysis was performed using one-way ANOVA with Dunnett’s multiple comparisons test (****p < 0.0001; ns, not significant). Sample size: *n* > 50 germlines per condition. Scale bar = 20 μm.

Thiostrepton has been reported to have an anti-cancer effect on various cancer cell lines by blocking the transcriptional activity of FoxM1 and reducing its expression, leading to apoptosis induction^12^. To assess the effect of Thiostrepton (Thio) in an *in vivo* context, we employed *C. elegans* as a model organism to investigate its impact on apoptosis at the whole-organism level. To this end, we treated the larval L4 stage worms with various dosages of Thio (from 100 µM to 180 µM) for 24 h and investigated the effect of this treatment on germline apoptosis induction. To quantify apoptotic cells following Thio treatment, we used a *C. elegans* strain expressing CED-1::GFP, a well-established reporter for apoptotic corpse engulfment. CED-1 is a transmembrane receptor that accumulates around apoptotic cell corpses, and its GFP-tagged form serves as a canonical marker for detecting apoptosis *in vivo*^18^. Treating *ced-1::gfp* worms with 150 µM and 180 µM doses of Thio resulted in a significant increase in apoptotic cell death in the germline of the developing *C. elegans*, confirmed by the accumulation of CED-1 protein around the dying cells (Fig. 1a). Analyzing the pachytene region of the Thio-treated germline using Differential Interference Contrast (DIC) optics highlighted the morphological features of apoptotic cell death that differentiate the corpses from healthy non-dying cells. These features include a button-like appearance under the DIC microscope due to their engulfment by the germline sheath cells and changes in the refractive index of the dying cells (Fig. 1a). Notably, treatment with 180 µM Thio induced the highest number of apoptotic cells compared to the untreated control (Fig. 1a and b). Our results indicate that a 24 hour exposure to 180 µM Thio triggers significant apoptosis in the *C. elegans* germline. Importantly, treatment with a range of Thio concentrations (5–180 µM) did not cause developmental arrest or observable changes in gross morphology (Suppl. Figure 1), suggesting that the compound selectively induces germline apoptosis without compromising overall organismal development.

### Thiostrepton-induced germ cell apoptosis depends on the core apoptotic machinery

**Fig. 2:**
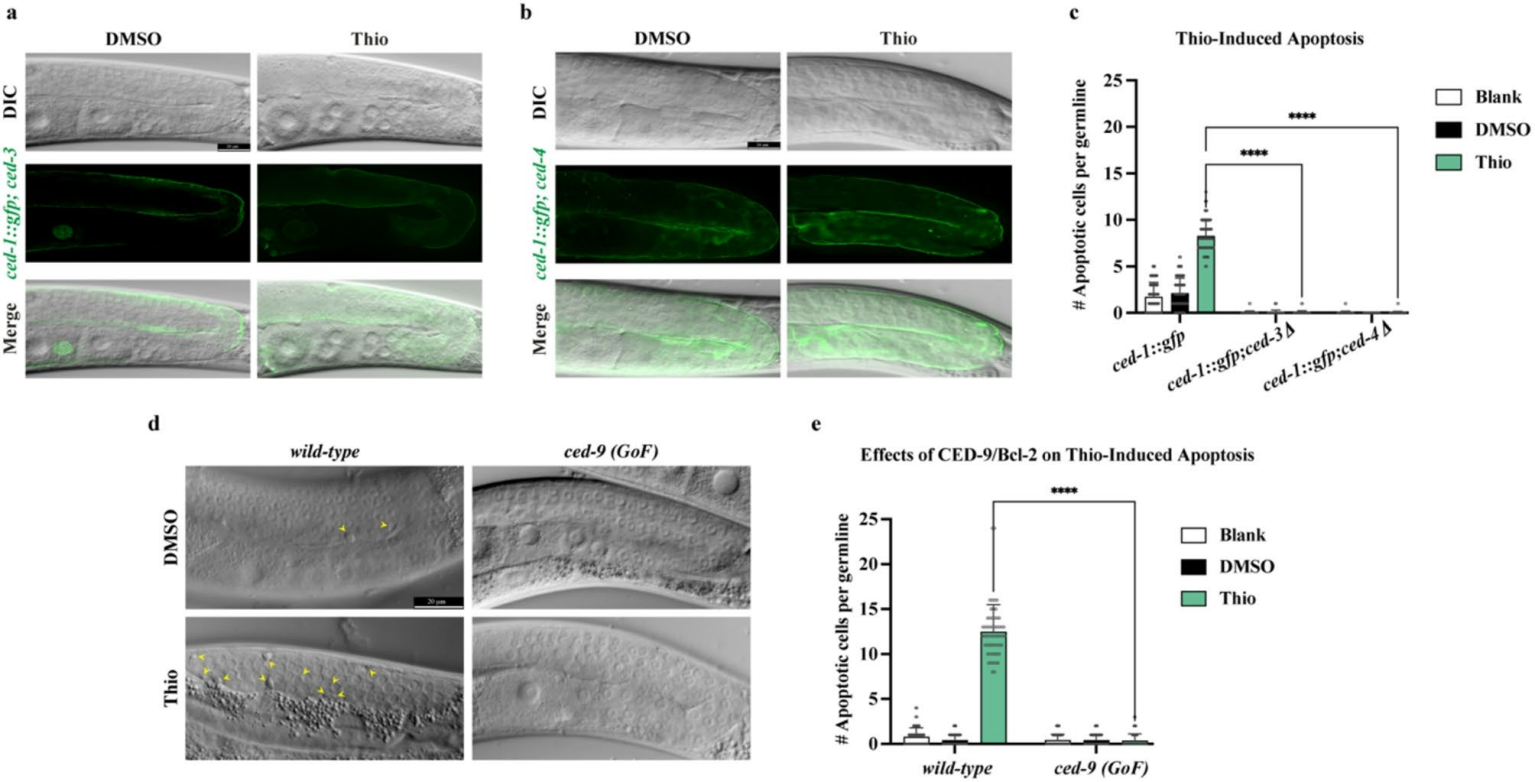
Thio-Induced Apoptosis Depends on CED-4/Apaf1 and CED-3/Caspase. **a** Representative DIC, GFP, and merged images of adult germlines from *ced-1::gfp; ced-3(n2452)* mutant worms treated with DMSO or 180 µM Thio for 24 h. No CED-1::GFP-positive apoptotic corpses are observed in Thio-treated worms, indicating that apoptosis induction is abolished in the absence of functional CED-3/caspase. Scale bar = 20 μm. **b** Representative germline images of *ced-1::gfp; ced-4(n1162)* mutant worms following DMSO or 180 µM Thio treatment. Similar to the *ced-3* mutant, Thio treatment fails to induce apoptotic corpses in the pachytene region of *ced-4(n1162)* mutants, confirming that CED-4/Apaf1 is also essential for Thio-induced germ cell apoptosis. Scale bar = 20 μm. **c** Quantification of apoptotic germ cell corpses per gonad in *ced-1::gfp*,* ced-1::gfp; ced-3(n2452)*,* and ced-1::gfp; ced-4(n1162)* worms following treatment with 180 µM Thio. Bars represent mean ± SEM. Statistical analysis was performed using one-way ANOVA with Dunnett’s multiple comparisons test (****p < 0.0001). *n* > 50 germlines were analyzed per condition. **d** Representative DIC images of germlines from wild-type and *ced-9(n1950 (GoF)* (gain-of-function) adult worms treated with DMSO or 180 µM Thio. Thio-treated wild-type germlines display distinct apoptotic corpses (yellow arrowheads), which are absent in the *ced-9 (GoF)* background, indicating that Thio-induced apoptosis is dependent to CED-9/BCL-2 function. Scale bar = 20 μm. **e** Quantification of germ cell corpses in wild-type and *ced-9 (n1950) (GoF)* mutants following Thio treatment. Bars represent mean ± SEM. Statistical significance was determined using one-way ANOVA (****p < 0.0001). *n* > 50 germlines were analyzed per condition.

During *C. elegans* development, a total of 131 cells are eliminated by apoptosis as part of the worm’s normal development. Nearly all of these developmental apoptotic events are executed by the core apoptotic machinery, which includes the BH3-only protein EGL-1, the BCL-2 homolog CED-9, the Apaf-1 ortholog CED-4, and the sole *C. elegans* caspase, CED-3. The executive CED-3 caspase is the most downstream component of the core apoptotic machinery in worms and is activated by its physical interaction with CED-4/Apaf1^19-23^. To functionally characterize the molecular mechanism of Thio-induced apoptosis, we aimed to measure the effect of 180 µM Thio on germ cells in various apoptotic-deficient mutants. To this end, we treated *ced-3(n2452)* and *ced-4(n1162)* mutants carrying the CED-1::GFP marker with 180 µM Thio for 24 hours. *ced-3(n2452)* contains a deletion in *ced-3*, and *ced-4(n1162)* carries a point mutation that abolishes their proapoptotic function. As expected, in both mutants, Thio treatment failed to induce apoptosis in the pachytene-stage germ cells (Fig. 2a, b, and c). To determine whether Thio-induced apoptosis depends on CED-9/BCL-2, we quantified apoptotic germ cell corpses in the *ced-9* gain-of-function (GoF) mutant following Thio exposure. Strikingly, Thio-induced apoptosis was completely abolished in the *ced-9* (GoF) background, indicating that CED-9 is essential for mediating Thio-triggered cell death in the germline (Fig. 2d and e).

## Thiostrepton-induced germ cell apoptosis is independent of DNA damage response and p53 function

**Fig. 3:**
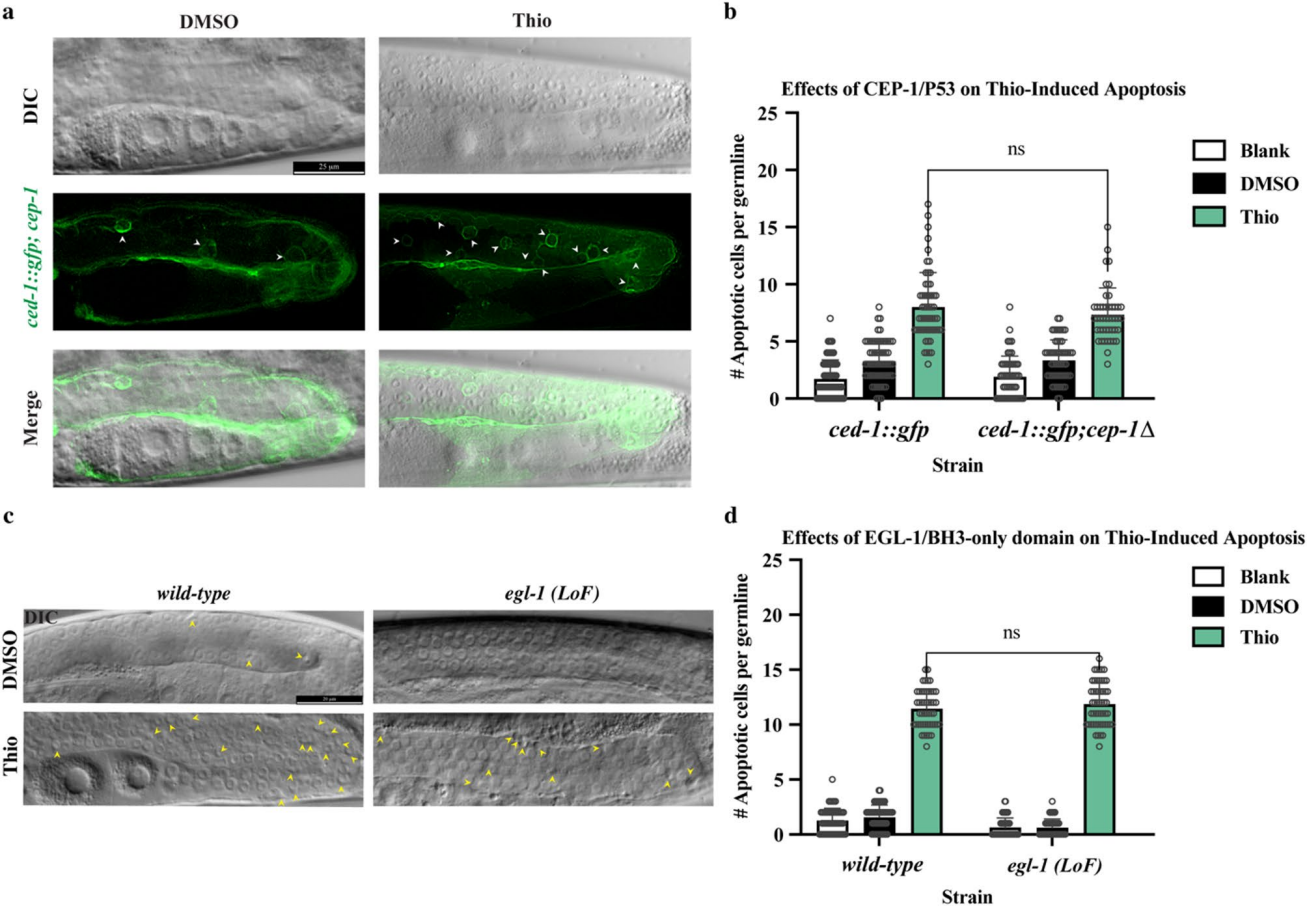
Influence of p53 Activity on Thio-Induced Apoptosis in *C. elegans* Mutants **a** Representative DIC, GFP, and merged images of adult germlines from *ced-1::gfp; cep-1(lg12501)* double mutant worms treated with DMSO or 180 µM Thio for 24 h. Thio induces apoptosis in the *cep-1* loss-of-function background, indicating that Thio-induced apoptosis is independent of CEP-1/p53 protein. Scale bar = 20 μm. **b** Quantification of apoptotic germ cell corpses per gonad in *ced-1::gfp*, and *ced-1::gfp; cep-1(lg12501)* double mutants following DMSO or 180 µM Thio treatment. Bars represent mean ± SEM. Statistical analysis was performed using one-way ANOVA with Dunnett’s multiple comparisons test; ns = not significant. *n* > 50 germlines per condition. **c** Representative DIC images of adult germlines from wild-type and *egl-1* (*LoF*) mutants treated with DMSO or 180 µM Thio. Apoptotic corpses (yellow arrowheads) are visible following Thio treatment in both genotypes. **d** Quantification of germ cell corpses in wild-type and *egl-1*(*LoF*) strains after DMSO or 180 µM Thio treatment. Bars represent mean ± SEM. No significant difference was observed in the absence of EGL-1, indicating that Thio-induced apoptosis occurs independently of EGL-1. Statistical analysis was performed using one-way ANOVA (ns = not significant). *n* > 50 germlines per condition.

In *C. elegans*, germ cell apoptosis occurs either as a physiological component of normal oogenesis and tissue homeostasis or in response to genotoxic stress via activation of conserved DNA damage response pathways mediated by the tumor suppressor protein CEP-1, the *C. elegans* ortholog of p53^24,25^. *C. elegans* CEP-1, the ancestral homolog of the mammalian p53, transcriptionally induces the BH3-only protein *egl-1* in response to genotoxic insult^26,27^. To test if germ cell apoptosis induction upon Thio treatment was mediated by the activation of the conserved DNA damage pathway and CEP-1/p53, we scored the apoptotic germ cells upon treatment with 180 µM Thio in both *ced-1::gfp; cep-1(Ig12501)*, and *egl-1(n1084n3082)* mutants. *cep-1(Ig12501)* contains a loss-of-function mutation that hinders CEP-1 capacity to trigger apoptosis upon DNA damage, while *egl-1* is a loss-of-function mutant that abolishes most DNA damage-induced cell death in the *C. elegans* germline. Interestingly, the *cep-1* and *egl-1* mutants exhibited apoptosis induction comparable to the wild-type strain, indicating that Thio induces apoptosis in the absence of functional *cep-1*/p53 and *egl-1*/ BH3-only protein (Fig. 3a, b, c, and d).

**Fig. 4:**
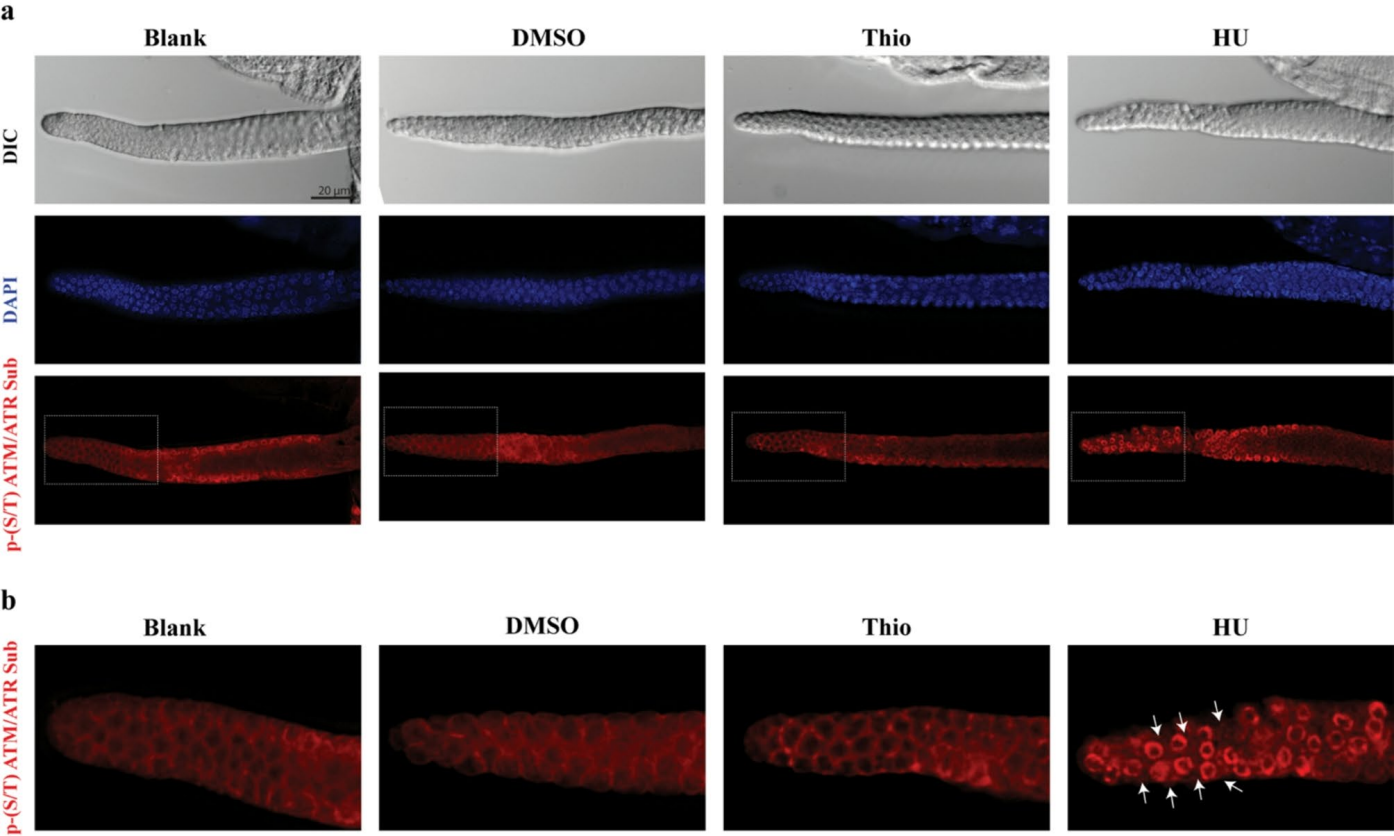
Thio Does Not Induce Activation of the ATM/ATR Pathway in *C. elegans* Germline. **a** Immunostaining of dissected germlines from worms treated under the following conditions: Blank (untreated), DMSO, 180 µM Thio, and 25 mM hydroxyurea (HU; positive control). Germlines were stained with a phospho-ATM/ATR substrate motif antibody (red), which specifically detects proteins phosphorylated on (S/T) QG motifs, substrates of ATM/ATR kinases. DAPI (blue) marks nuclei. As expected, HU-treated worms displayed robust focal activation of ATM/ATR signaling, while no such activation was observed under Thio or DMSO conditions. **b** Zoomed-in of the mitotic region from panel a (dashed box). White arrowheads indicate distinct nuclear phospho-ATM/ATR-positive staining observed only in 25 mM HU-treated samples; however, no such phospho-ATM/ATR substrate staining was detected in Blank, DMSO, or Thio-treated germlines. Scale bars = 20 μm.

Although the apoptotic phenotype mediated by Thio treatment was independent of the p53 function, we aimed to further confirm that this treatment did not induce DNA damage response (DDR) or genomic instability. Therefore, to test whether Thio-induced apoptosis involves activation of the canonical DNA damage response pathway, we assessed the phosphorylation status of ATM/ATR substrates using an antibody that specifically recognizes the conserved phospho-(S/T) QG motif^28-30^. Immunostaining was performed on dissected germlines from Thio-treated worms and compared to untreated controls. As a positive control, worms were treated with 25 mM hydroxyurea (HU), a known genotoxic agent that robustly activates the DNA damage checkpoint. As expected, HU treatment led to a strong phospho-(S/T) QG signal in the germline, indicative of ATM/ATR activation. In contrast, no detectable phospho-(S/T) QG signal was observed in Thio-treated samples, indicating that Thio does not activate ATM or ATR kinases (Fig. 4a and b). To confirm further that Thio treatment did not activate the canonical DNA damage response pathway, we investigated the activity of Checkpoint Kinase 1 (CHK-1). Upon genotoxic stress, CHK-1 is phosphorylated on Ser 345 through the activity of the ATM protein kinase. We therefore stained the germline of the Thio-treated worms with the antibody against CHK-1 p^Ser 345^. In line with our previous data, we observed no increase in CHK-1 phosphorylation upon Thio treatment (Suppl Fig. 2a).

To determine whether Thio treatment induces single- or double-strand DNA breaks, we examined the formation of RPA-1 foci in germ cells. RPA-1 is a key protein involved in multiple aspects of DNA metabolism, including replication, repair, and checkpoint activation^31,32^. The accumulation of RPA-1 foci on chromatin is a well-established marker of DNA damage and a critical upstream event in the activation of the ATM/ATR-dependent DNA damage checkpoint pathway^28^. To test this, we used the *C. elegans* transgenic strain SSM473, which expresses RPA-1::GFP, and exposed worms to 180 µM Thio. We then quantified RPA-1 foci formation in the germline. As expected, Thio-treated animals showed no significant increase in RPA-1 foci compared to controls, indicating that Thio does not induce detectable levels of genomic instability (Suppl. Figure 2b). Taken together, the absence of RPA-1 foci formation, the lack of CHK-1 phosphorylation, and the negative phospho-ATM/ATR substrate motif staining strongly support the conclusion that Thio-induced apoptosis in *C. elegans* occurs independently of the canonical DNA damage checkpoint pathway.

### Thiostrepton induces apoptosis in the somatic cells of *C. elegans*

To test if Thio also induces somatic cell death and its effect is not limited to germ cells, we measured apoptosis induction in the developing worms. To this end, we took advantage of *ced-1(e1735)*, a loss-of-function mutant strain that is defective in the elimination of apoptotic cells, making the apoptotic cells persist throughout the development. Interestingly, the Thio-treated Larvae staged 1 (L1) worms showed a significantly increased number of apoptotic cells in the pharyngeal region compared to the control (Suppl. Figure 3a and b). These findings suggest that Thio treatment also induces somatic apoptosis and generally exerts its effect at the level of the core apoptotic machinery.

### Thiostrepton treatment does not increase the level of reactive oxygen species

**Fig. 5:**
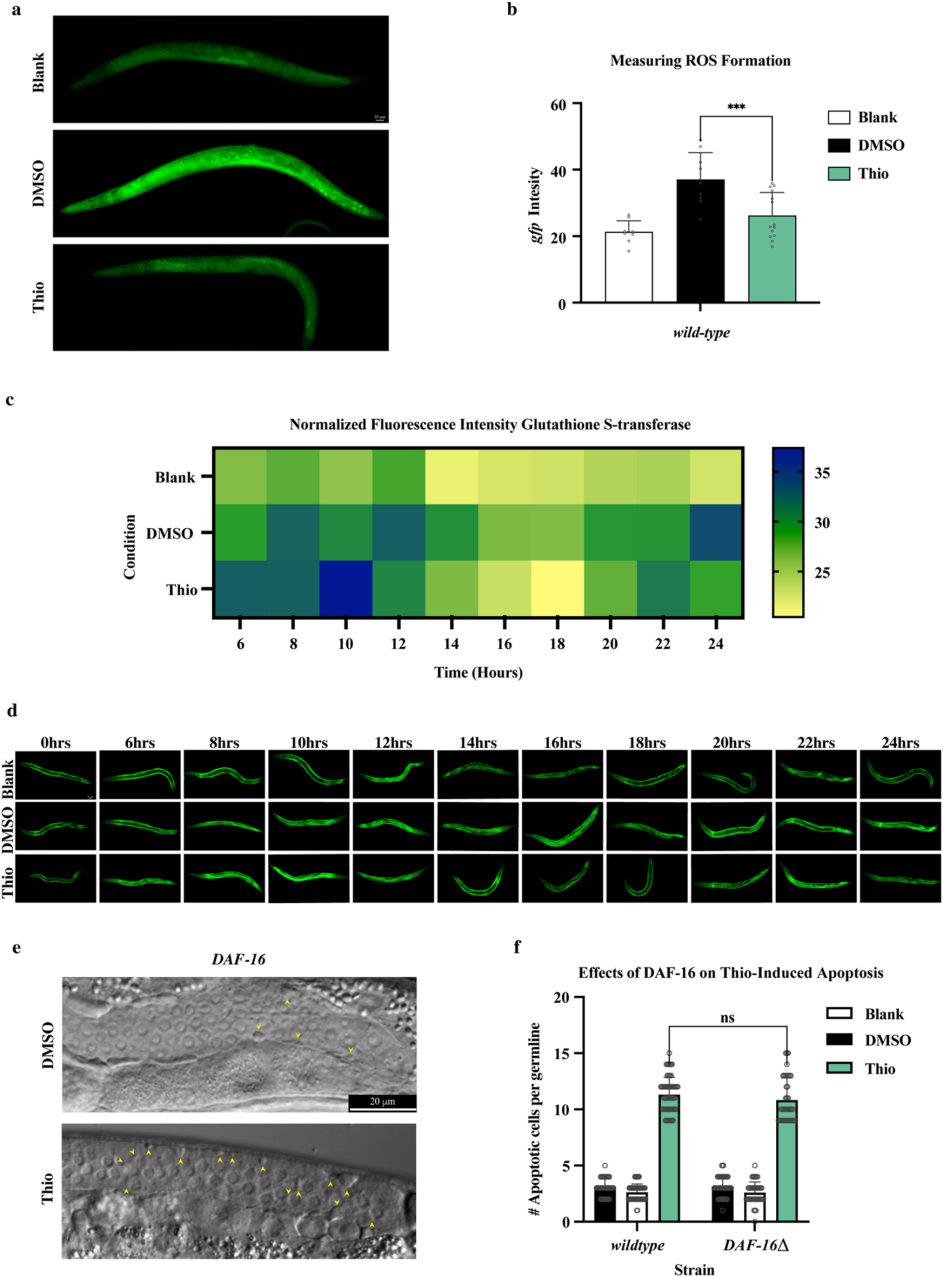
Measuring the Generation of Reactive Oxygen Species Upon Thio Treatment. **a** Representative fluorescent image of wild-type worms stained with CellROX Green, comparing untreated (Blank), DMSO-treated, and 180 µM Thio-treated conditions. Thio-treated worms do not show significant changes in the GFP fluorescence intensity, indicating that Thio does not induce ROS formation. Scale bar = 20 μm. **b** Quantification of ROS levels based on GFP fluorescence intensity in wild-type worms following the treatments shown in a. Fluorescence was measured using ImageJ and statistically analyzed in GraphPad Prism. Bars represent mean ± SEM; ***p < 0.001 by one-way ANOVA. **c** Heatmap showing normalized GST-4::GFP fluorescence intensity in worms treated with Blank, DMSO, or Thio, measured every 2 h from 6 to 24 h post-treatment. Fluorescence intensity was quantified using ImageJ, normalized to background, and used to visualize dynamic GST-4 response across conditions. **d** Representative images of GST-4::GFP-expressing worms under each condition (Blank, DMSO, Thio) at each time point (6–24 h). These images illustrate the visual GST-4::GFP expression patterns corresponding to the quantified data shown in (c). **e** DIC images of adult germlines from *daf-16(mu86)* mutant worms treated with DMSO or Thio. Yellow arrowheads mark apoptotic cell corpses in the pachytene region. Scale bar = 20 μm. *n* > 50 germlines per condition. **f** Quantification of apoptotic germ cell corpses per gonad in wild-type and *daf-16(mu86)* mutant worms treated with Blank, DMSO, or Thio. Bars represent mean ± SEM. No statistically significant difference was detected between the wild type and *daf-16(mu86)* mutants. Statistical analysis was performed using one-way ANOVA; ns = not significant. *n* > 50 germlines per condition.

It has previously been reported that Thio is capable of elevating intracellular reactive oxygen species (ROS) levels in various cell lines, and this elevation in ROS is believed to be linked to apoptotic induction^11,33^. To test if Thio treatment induced ROS production in *C. elegans* and if an increase in ROS could be potentially linked to apoptosis induction, we quantified ROS levels in the wild-type worms with and without Thio treatment. Surprisingly, we observed no significant changes in ROS production upon Thio treatment (Fig. 5a and b). Our results demonstrate that a 24 hour Thio treatment does not increase intracellular ROS. To our surprise, Thio treatment reduced ROS elevation in the DMSO control sample to some degree. Several studies have shown that DMSO treatment leads to increased ROS production in cell cultures^34^. Additionally, we evaluated the expression level of the member of the Glutathione S Transferase family, GST-4, a key protein involved in the cellular response to oxidative stress, upon Thio treatment across multiple time points (6–24 h). Previous reports have shown that *gst-4::GFP* is a robust reporter to measure cellular oxidative stress^35^. Although a slight increase in the GST-4::GFP signal was observed between 10 and 18 h compared to the control, the overall elevation of the GFP signal was minimal and fluctuated stochastically across most of the time points. Altogether, there were no significant differences in intensities across the conditions and time points upon Thio treatment (Fig. 5c and d).

### Thiostrepton-induced apoptosis is independent of FOXO/DAF-16

FoxM1 is an oncogenic transcriptional factor associated with various types of cancer^36-38^. There are over 40 members of forkhead transcriptional factors that fall into 19 subfamilies according to their sequence similarities, which are evolutionary and highly conserved from *C. elegans* to humans^39^. Thio has been reported to inhibit FOXM1 transcriptional factor in a p53-dependent manner. To test if Thio-induced apoptosis in *C. elegans* involves the conserved FOXO transcription factor DAF-16, which mediates stress response, we examined the apoptotic response to Thio in *daf-16* loss-of-function mutants. Surprisingly, Thio treatment induced apoptosis in *daf-16* mutants at levels comparable to wild-type controls (Fig. 5e, f). These results indicate that Thio-induced apoptosis is independent of the DAF-16/FOXO axis, further confirming that Thio acts through alternative or downstream mechanisms unrelated to classical oxidative stress responses.

## Discussion

This study revealed the proapoptotic effect of thiostrepton (Thio) in *C. elegans*. We have demonstrated that the exposure of *C. elegans* to 180 μM of Thio induces caspase-dependent apoptosis at the level of CED-9/BCL-2. We have further shown that, unlike in mammalian cells, Thio-induced apoptosis in *C. elegans* is independent of the CEP-1/p53 function and unrelated to the DNA damage response. Also, we have clearly shown that the exposure of Thio to *C. elegans* does not lead to oxidative stress, although this finding contrasts with that reported in studies on human cells. Using various mutants of the core apoptotic machinery, we have shown that treating *C. elegans* with 180 μM of Thio results in apoptotic cell death. Treating *C. elegans* with Thio at various concentrations for 24 h leads to germ cell apoptosis that is dependent on the activity of the caspase/CED-3. We have systematically investigated the proapoptotic effect of Thio in various mutants. As expected, the *C. elegans* counterpart of Apaf1, CED-4, is essential for Thio-induced apoptosis, as a loss-of-function mutation in *ced-4* abrogates the apoptotic phenotype. Interestingly, the apoptotic phenotype observed with Thio treatment was dependent on the activity of the anti-apoptotic gene, CED-9/BCL-2. Thio failed to induce apoptosis in the germline of the CED-9 gain-of-function mutants, suggesting that the effect of Thio is most likely upstream or at the level of CED-9/BCL-2 protein. This is in line with previous reports on the anti-cancer effect of Thio in melanoma cell lines. Treating melanoma cells with Thio induces apoptotic death that is inversely correlated with the antiapoptotic Mcl-1 expression level^40^.

Previous research in human cancer cell lines has established that Thio induces the accumulation of reactive oxygen species (ROS), which play a critical role in mediating its pro-apoptotic effects. For example, in recent studies using colorectal cancer models, Thio-induced endoplasmic reticulum (ER) stress and apoptosis were significantly attenuated by co-treatment with the antioxidant N-acetylcysteine (NAC), underscoring the central role of ROS in its cytotoxic mechanism and apoptosis. Additionally, it is well studied that Thio induces apoptosis in B-precursor acute lymphoblastic leukemia cells, and this effect is highly reduced by co-treatment with the antioxidant NAC, supporting a ROS-dependent mechanism of action, an effect notably absent in our *C. elegans* model^11^.

More recent studies have shown that Thio induces DNA damage response and activates the tumor suppressor protein p53^15^. To understand the mechanism of Thio-induced apoptosis in an *in vivo* setting, we aimed to test if Thio could activate the DNA damage response and p53 induction. The antiproliferative and proapoptotic activity of Thio *in vitro* is well-established, and previous studies have reported its various modes of action. It has been shown that Thio physically interacts with and inhibits FoxM1. Thio treatment leads to the downregulation of FoxM1 at the RNA level, decreasing the proliferation rate in B-precursor acute lymphoblastic leukemia (B-pre-ALL)^1^. Other studies have suggested that Thio induces cell death by increasing the bax/bcl-2 ratio, a cell death switch, leading to the release of cytochrome c and activation of caspases^11^. On the other hand, treatment of worms carrying a loss-of-function mutation of *cep-1*/p53 with Thio did not influence the apoptotic response, indicating that Thio-induced apoptosis is independent of CEP-1/p53.

In addition, our findings provide compelling evidence that Thio induces apoptosis in *C. elegans* through a mechanism that is independent of the canonical DNA damage response pathway. Unlike in mammalian systems, where Thio has been shown to induce DNA damage and activate p53 via generation of ROS and activating checkpoint kinases such as ATM and ATR, our *in vivo* data in *C. elegans* reveal a distinct mode of action. Specifically, we show that Thio treatment does not lead to phosphorylation of ATM/ATR substrates, nor does it induce downstream markers such as phosphorylation of the checkpoint kinase protein, CHK-1. We analyzed the chromatin-associated RPA-1 foci formation upon Thio treatment but observed no significant increase. These results strongly suggest that Thio-induced apoptosis bypasses the classical DNA damage signaling axis. In contrast to previous reports in mammalian systems where Thio induces apoptosis through ROS generation and FOXM1 suppression in a p53-dependent manner, our findings in *C. elegans* reveal a markedly different mode of action. Specifically, we observed that Thio-induced apoptosis occurs independently of DAF-16, the sole *C. elegans* ortholog of FOXO and a central regulator of oxidative stress response, metabolism, and longevity. Thio treatment in *daf-16* mutant worms resulted in levels of apoptosis comparable to wild-type controls, indicating that *DAF-*16 is not required for the Thio pro-apoptotic effect. Importantly, to directly assess ROS involvement, we employed two complementary methods: the CellROX®  ROS detection assay and a GST-4::GFP reporter strain, a well-established transcriptional sensor for oxidative stress. To our surprise, in both assays, Thio treatment did not lead to detectable ROS accumulation, further supporting the conclusion that Thio-induced apoptosis in *C. elegans* is ROS-independent. These findings contrast with human cancer cell models, where Thio-induced apoptosis is often tightly linked to oxidative stress. Together, our data highlight a distinct, *in vivo* apoptotic mechanism for Thio that bypasses the classical ROS and FOXO/DAF-16 stress response axis. This divergence may reflect species-specific differences in redox homeostasis, DNA damage sensing, or drug metabolism, but it also raises the intriguing possibility that Thio can engage the core apoptotic machinery directly, independent of upstream genotoxic stress. Although DAF-16 is the predominant Forkhead transcription factor in *C. elegans*, it is important to note that the worm genome does not encode a homolog of FOXM1, the mammalian oncogenic FoxM subfamily member. Nevertheless, further investigation into the potential roles of other Forkhead box–containing transcription factors such as PHA-4 (FOXA ortholog), which is involved in pharyngeal development, and UNC-130 (FOXC ortholog), which regulates cell fate specification, may provide additional insights into the mechanism of Thio induced apoptosis in *C. elegans* and in an *in vivo* setting. Exploring whether these factors contribute to Thio-induced cell death could reveal novel, context-specific regulatory mechanisms.

Altogether, our genetic and cytological data indicate that the apoptotic phenotype mediated by Thio is unrelated to the DNA damage response. All in all, in the context of cancer therapy, our findings could be particularly relevant in tumor cells with defective checkpoint signaling or p53 inactivation. Therefore, our *C. elegans* data may have broader implications for understanding how Thio or similar compounds could trigger apoptosis in checkpoint-compromised systems.

## Materials and methods

### *C. elegans* strains and maintenance

*C. elegans* strains were maintained at 20°C on Nematode Growth Media (NGM) plates seeded with *E. coli* (OP50) bacteria as described previously^41^. *C. elegans* strains used in this study: Bristol N2 (wildtype), MD701: *bcIs39 [lim-7p::ced-1::GFP + lin-15(+)]*, EPD077: *cep-1(Ig12501);[lim-7p::ced-1::GFP + lin-15(+)]*, KX84: *ced-3(n2452) IV; bcIs39 V*, CB3203: *ced-1(e1735)I*, KX89: *ced-4(n1162) III; bcIs39 [lim-7p::ced-1::GFP + lin-15(+)]*, SSM473: *rpa-1(iow89[GFP11::rpa-1])II; iowSi8II; unc-119(ed3)III*, MT4770: *ced-9 (n1950) III*, CF1038: *daf-16 (mu86)*, MT8735: *egl-1(n1084n3082)*, and CL2166: *dvIs19 [(pAF15) gst-4p::gfp::nls]*.

### Thiostrepton treatment

Synchronized L4-stage worms were exposed to Thio using a liquid culture assay in 96-well plates. Thio was dissolved in dimethyl sulfoxide (DMSO) to a stock concentration of 10 mM and diluted to final working concentrations of 100, 120, 150, or 180 µM in M9 buffer supplemented with *E. coli OP50*. Each treatment well contained 30 worms in a total volume of 80 µL. DMSO concentration was standardized across all conditions. The plates were incubated on a shaker at 180 rpm for 24 h at room temperature. After treatment, worms were transferred to unseeded NGM plates and prepared for downstream analyses.

### Scoring of apoptotic cells

Apoptotic cells were quantified using a Leica DM6 B 3D-Thunder Imager equipped with DIC optics. For germline apoptosis scoring, adult worms expressing the *ced-1::gfp* were immobilized on 3% agarose pads using 50% levamisole from 1 M levamisole. Samples were visualized at 40× magnification, and apoptotic cell corpses were identified either by their button-like morphology under DIC or by the accumulation of CED-1::GFP signal around the apoptotic nuclei in the pachytene region of the gonad^38^. For somatic apoptosis analysis, L1-stage progeny of treated worms were analyzed in the *ced-1(e1735)* mutant background, which impairs apoptotic corpse clearance and allows detection of persistent pharyngeal corpses. These were imaged and scored under DIC optics at 40× magnification. Apoptosis data were compiled in tabulated form and statistically analyzed using GraphPad Prism.

### Immunostaining and microscopy

Germline extraction and immunostaining were performed as previously described^19,32^. In brief, following a 24 hour treatment with 180 µM Thiostrepton or DMSO, adult worms were transferred to unseeded NGM plates for 10–15 min to remove residual bacteria. Approximately 8–10 worms were then transferred into 8 µL of dissection buffer (0.2 mM levamisole, 0.2% Tween-20 in egg buffer) placed on a 22 × 22 mm coverslip. Germlines were dissected by severing the worms near the pharynx or tail using a 23-gauge needle. The released germlines were immediately fixed using 2% paraformaldehyde (in egg buffer with 0.2% Tween-20), then transferred onto poly-L-lysine–coated slides and incubated at room temperature for 5 min. Slides were flash-frozen in liquid nitrogen and freeze-cracked by removing the coverslip. Samples were fixed in ice-cold acetone: methanol (1:1, v/v) for 10 min, then permeabilized by three washes in PBS with 1% Triton X-100, each for 10 min. Blocking was performed with 1% BSA (in PBS-Tween 0.1%) for 20 min. Samples were incubated overnight at 4°C with 40 µL of primary antibody solution. Slides were then washed in PBS-Tween 0.1% for 30 min (refreshing every 10 min), followed by 2 h incubation at room temperature with the secondary antibodies. Nuclei were counterstained with 0.5 µg/mL DAPI. Following final washes, samples were mounted using Vectashield antifade mounting medium (H-1000) and sealed with 22 × 22 mm coverslips.

The following primary antibodies were used in this study: rabbit monoclonal anti-phospho-CHK-1 (Ser345) (Thermo Scientific, MA5-15145) at a dilution of 1:200, and Anti-Phospho-ATM/ATR substrate motif [(pS/pT) QG] (Cell Signaling Technology, 6966, 1:200). For the secondary antibodies, Alexa Fluor^®^ 568–conjugated donkey anti-rabbit IgG (Abcam, ab175470) secondary antibody dilution of 1:200 dilution were used.

Microscopy was performed using a Leica DM6 B 3D-Thunder Imager equipped with a Leica K5 sCMOS camera and 40× dry objective. The Small Volume Computational Clearing (SVCC) module was used to reduce out-of-focus background and enhance image clarity. Z-stacks (~ 0.3 μm intervals) were acquired and visualized using Leica Application Suite X (LAS X v3.8.1.26810). Images were exported in 16-bit format.

### Analyses of oxidative stress

Reactive Oxygen Species (ROS) levels were assessed using the CellROX^®^ Green Reagent (C10444, Invitrogen, USA), which fluoresces upon oxidation and DNA binding. Following Thiostrepton treatment (as described above), worms were washed three times with M9 buffer to remove residual bacteria and treatment solution. Worms were then fixed in 2% paraformaldehyde for 30 min at room temperature on a rotating platform (300 rpm), followed by three washes with 1× PBS. Fixed samples were incubated in 5 µM CellROX Green solution (diluted in 1× PBS) for 1 h at room temperature in the dark with gentle agitation. After staining, samples were washed three additional times with 1× PBS and mounted on 3% agarose pads for imaging.

Fluorescence imaging was performed using a Leica DM6 B 3D-Thunder Imager equipped with a GFP filter and 20× objective lens. Images were acquired under standardized exposure conditions, and fluorescence intensity was quantified using ImageJ (NIH). ROS signal values were normalized to background fluorescence to assess relative oxidative stress levels.

### Statistical analysis

Statistical analysis and graph generation were performed using GraphPad Prism 10 for macOS, Version 10.1.0 (264), GraphPad Software, San Diego, California, USA. The significance level was set at p= 0.05. For all experiments, at least three independent biological replicates were used to assess statistical significance unless otherwise specified. For comparisons between control and treated groups, ordinary two-way ANOVA was used, and Dunnett’s or Tukey’s post-hoc test was applied to assess pairwise differences. For unpaired comparisons involving two groups, Welch’s t-test was used, and an F-test was applied to assess variance differences between groups. Details of specific statistical tests used for each dataset, including sample sizes and p-values, are provided in the corresponding figure legends.

## Electronic supplementary material

Below is the link to the electronic supplementary material.

Supplementary Material 1

## Data Availability

The datasets used and/or analyzed during the current study are available from the corresponding author on reasonable request.
