# Supplementary material for: Thiostrepton induces apoptotic cell death at the level of BCL-2/CED-9 in C. elegans
Source: Sci Rep. 2025 Jul 8;15:24415. doi: 10.1038/s41598-025-09446-5 (PMC12238647; doi:10.1038/s41598-025-09446-5)
Supplement: Supplementary file 1 — Supplementary Material 1 [file 41598_2025_9446_MOESM1_ESM.pdf]

Thiostrepton Supplementary – Figures and Legends

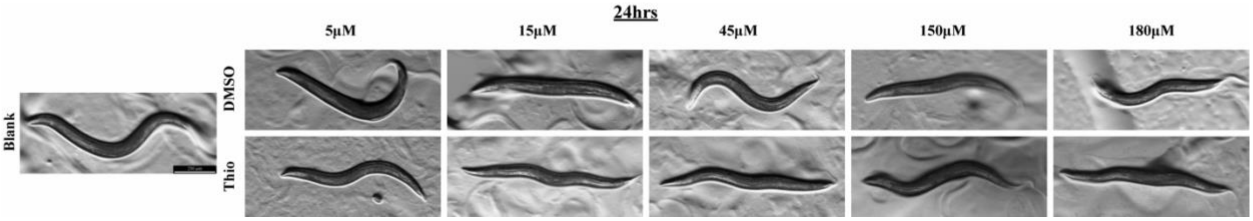

Suppl. Fig. 1 The Effects of Thio on the Development of *C. elegans*. Representative DIC images of adult *C. elegans* after 24-hour exposure to increasing concentrations of Thio (5, 15, 45, 150, and 180 µM), compared to DMSO-treated and untreated (Blank) controls. No observable defects in body morphology or developmental arrest were evident across the tested concentrations, indicating that Thio does not cause overt toxicity under these conditions. Scale bar = 230 µm.

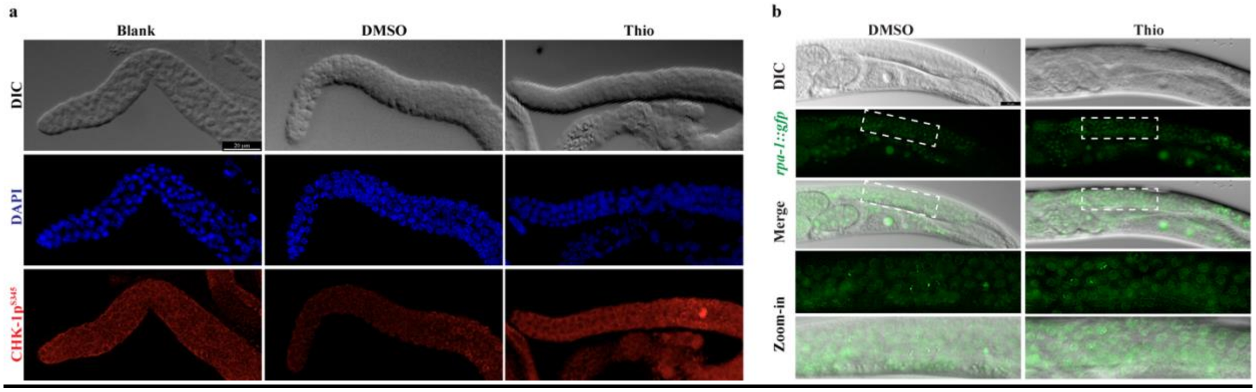

Suppl. Fig. 2 Effects of Thio Treatment on DNA Damage Response in *C. elegans* Germline. a Immunofluorescence images of dissected germlines from wild-type worms treated with Blank, DMSO, or Thio, stained with anti-phospho-CHK1 (Ser345) antibody (red) and DAPI (blue). Phosphorylation of CHK-1 on Ser^{345} is a hallmark of ATM/ATR-mediated DNA damage response. No elevation in phospho-CHK-1 signal was detected in Thio-treated samples, indicating that the DNA damage checkpoint is not activated by Thio exposure. **b** Representative DIC and fluorescence images of germlines from the *rpa-1::gfp* transgenic strain (*SSM473*) treated with DMSO or 180 µM Thio for 24 hours. RPA-1::GFP foci, indicative of DNA replication stress or DNA damage, were quantified in the pachytene region. No increase in RPA-1 foci was observed in Thio-treated worms. Zoomed-in regions highlight foci localization on the chromatin. Scale bar = 20 µm.

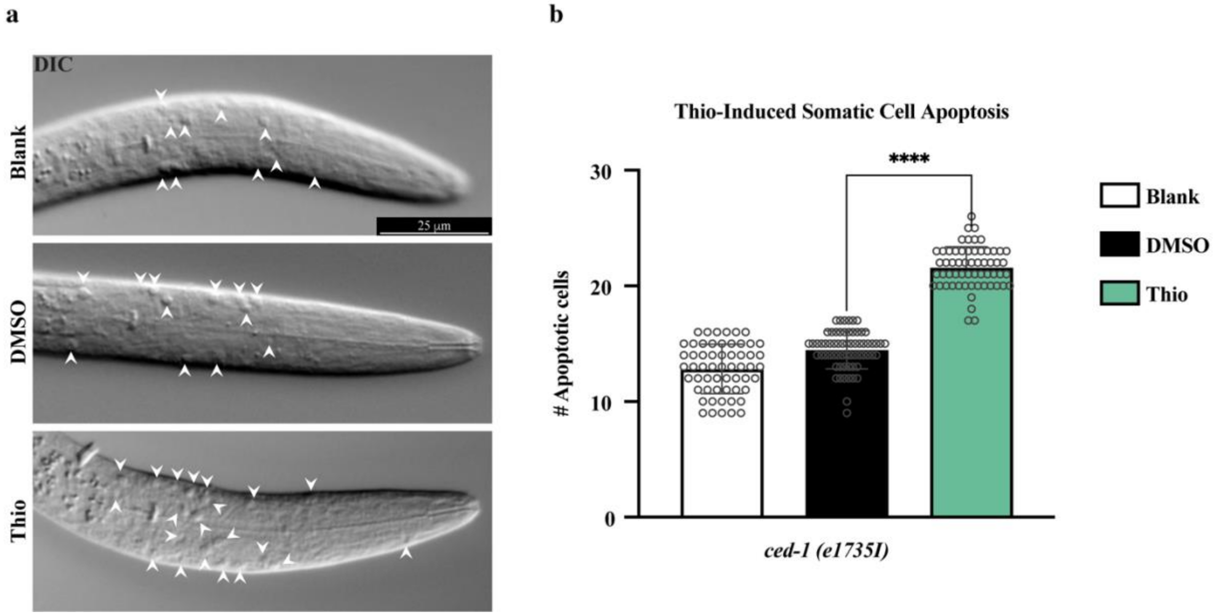

Suppl. Fig. 3 Impact of Thio on the Induction of Apoptosis in L1 stage worms

a representative DIC image showing the pharyngeal region of L1-stage *ced-1(e1735)* mutant worms treated with Blank, DMSO, or 180 μM Thio for 24 hours. Persistent apoptotic cells are visible as dense button-like structures marked by white arrowheads. **b** Quantification of apoptotic cells in the pharyngeal region of L1-stage worms following treatment. Bars represent the mean ± SEM. Thio treatment significantly increased the number of apoptotic cells compared to DMSO. Statistical analysis was performed using one-way ANOVA (****$p < 0.0001$). $n > 50$ worms per condition.
